# CD30‐positive lymphomatous (nonleukemic) variant of adult T‐cell leukemia/lymphoma, HTLV‐1 associated

**DOI:** 10.1002/ccr3.3092

**Published:** 2020-07-20

**Authors:** Mark Podberezin, Aliyah R. Sohani

**Affiliations:** ^1^ Department of Anatomic Pathology Lahey Hospital and Medical Center Burlington MA USA; ^2^ Department of Pathology Massachusetts General Hospital Boston MA USA

**Keywords:** adult T‐cell leukemia/lymphoma, CD30, HTLV‐1, lymphomatous variant

## Abstract

Nonleukemic variant of HTLV‐1‐associated adult T‐cell leukemia lymphoma (ATLL) is a rare variant, and herein, we describe a case with strong and diffuse positivity of neoplastic cells for CD30. Even though ATLL is aggressive entity with poor prognosis, in our case, there was very good clinical response achieved with brentuximab‐containing regimen. Therefore, HTLV‐1‐associated ATLL can be included in the differential diagnostic approach of CD30‐positive lymphoproliferative disorders.

## Graphical abstract

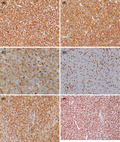

A 38‐year‐old previously healthy woman of Haitian origin presented with splenomegaly and extensive lymphadenopathy involving cervical, supraclavicular, axillary, mediastinal, hilar, para‐esophageal, para‐aortic, mesenteric, and inguinal lymph nodes. The largest lymph node measured 2.9 cm in size, and PET‐CT scan showed maximum SUV of approximately 32 mSv. No skin lesions were identified. Serum calcium was normal. Peripheral blood counts were within normal range, and no circulating abnormal lymphoid cells were seen.

The patient underwent a biopsy of a cervical lymph node. The lymph node architecture was effaced by an atypical infiltrate of large pleomorphic lymphoid cells with moderate eosinophilic cytoplasm, irregular nuclei, coarse to vesicular chromatin, and occasional prominent nucleoli (Figure 1A,B) amid a background of rare small lymphocytes and histiocytes.

**Figure 1:**
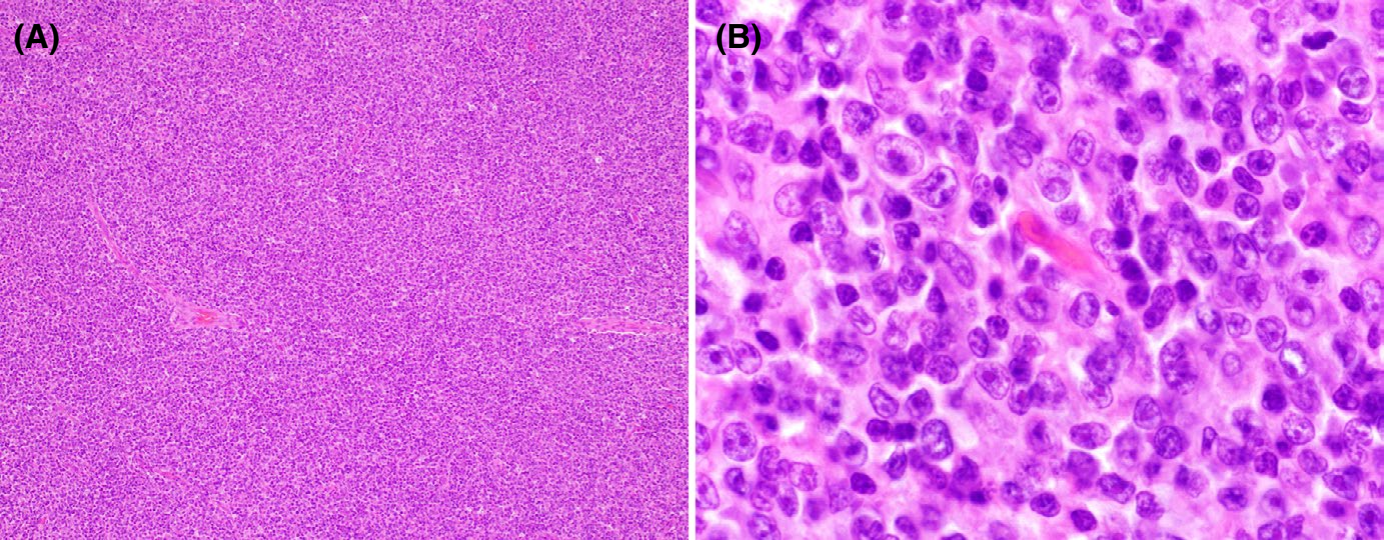
Cervical lymph node excisional biopsy. (A) low‐power H&E (20×, 200× total magnification); (B) high‐power H&E (100×, 1000× total magnification)

By immunohistochemistry, neoplastic cells were positive for CD3 (Figure 2A), CD4 (Figure 2B), CD25 (Figure 2C), and negative for CD7 (Figure 2D), positive for CD2, CD5, and Bcl‐2 (not shown). In addition, tumor cells were strongly positive for CD30 (Figure 2E) and PD1 (Figure 2F), CD8, CD10, CD20, Bcl‐6, ALK‐1, granzyme B, and CXCL‐13. Neoplastic cells showed 60% Ki67 proliferation index. CD21 highlighted rare small residual follicular dendritic cell meshworks. Immunohistochemical stains for CD68 and CD138‐stained admixed histiocytes and plasma cells, respectively. Flow cytometry showed an abnormal T‐cell population with expression of CD4, CD2, CD5, moderate CD3, and lack of CD7.

**Figure 2:**
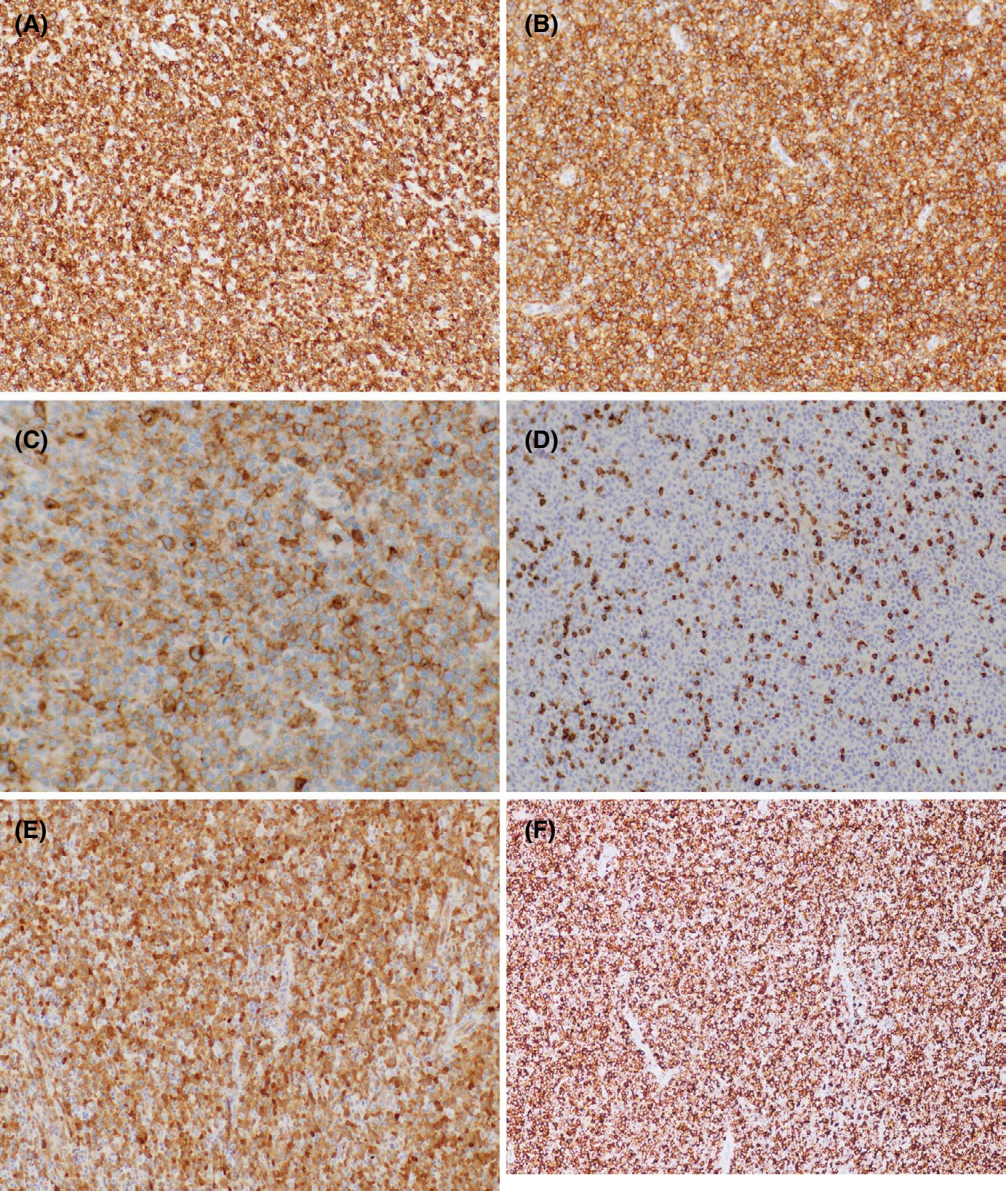
Cervical lymph node biopsy, immunohistochemical studies. (A) CD3 (20×, 200× total magnification), (B) CD4 (20×, 200× total magnification), (C) CD25 (20×, 200× total magnification), (D) CD7 (20×, 200× total magnification), (E) CD30 (40×, 400× total magnification), (F) PD1 (CD279) (10×, 100× total magnification)

Serologic tests showed anti‐human T‐cell leukemia virus (HTLV)‐1/2 antibodies with subsequent Western blot confirmation of HTLV‐1 infection. In this context, the histopathologic and immunophenotypic findings were consistent with adult T‐cell leukemia/lymphoma (ATLL), HTLV‐1 associated.

Staging bone marrow evaluation did not demonstrate involvement by lymphoma. Evaluation of cerebrospinal fluid showed no abnormal cells by cytology or flow cytometry. The patient began treatment with brentuximab, cyclophosphamide, adriamycin, vincristine, etoposide, and prednisone (BvCHEP) with intrathecal methotrexate with significant improvement in adenopathy after one cycle. Treatment is planned for a total of four cycles followed by allogenic stem cell transplant.

HTLV‐1 retrovirus has been present since ancient times.^1^ Its prevalence is very low (<0.1%) in the general population but exceeds 1% in certain areas of Caribbean, Pacific, and Africa.^2^ HTLV‐1 has a particularly high tropism for CD4 lymphocytes. Via binding of HTLV‐1 envelope surface proteins to CD4 surface receptors and subsequent fusion with a target cell, viral RNA is delivered into the cytoplasm, converted by reverse transcriptase into double‐stranded DNA which, in turn, is shuttled into the nucleus and is incorporated into the T‐cell genome as provirus, which can be detected by quantitative PCR in peripheral blood mononuclear cells.^3^

The major difference between HIV and HTLV‐1 is the fact that the latter stimulates mitosis of target CD4 T cells rather than viral replication.^2^ That is why, in contrast to HIV infection, patients with HTLV‐1 infection have normal or high, rather than low, CD4 counts. In addition, viral transmission between persons occurs through infected cell transfer and not by cell‐free virus. Major mechanisms of infectivity are breast feeding, blood transfusion, and sexual transmission.^3^ It has been estimated that in HTLV1‐endemic areas, 15%‐25% of children of infected mothers are also infected by the virus.^4^ Major diagnostic essays of HTLV‐1 infection include initial serum enzyme‐linked immunosorbent assay‐based screening test, with subsequent confirmation by Western blot.

All HTLV‐1‐related disorders are broadly classified into neoplastic, inflammatory, and infectious. Neoplastic HTLV‐1 associated disorder is represented by ATLL, and examples of infectious and inflammatory HTLV‐1 related entities include, but are not limited to, tropical spastic paraparesis (also known as HTLV‐1‐associated myelopathy), dermatitis, arthritis, uveitis, and myositis.

The 2017 WHO Classification of Tumours of Haematopoietic and Lymphoid Tissues separates four clinical variants of ATLL: smoldering, chronic, acute, and lymphomatous.^5^, ^6^ Recently, extranodal primary cutaneous variant of ATLL has been described.^5^ In contrast to most described cases of ATLL, our patient presented with isolated lymphadenopathy, without involvement of peripheral blood, bone marrow, or skin. Therefore, it is best classified as the lymphomatous type of ATLL.

Strong and diffuse expression of CD30, as seen in our case, is an unusual finding in ATLL. However, Takeshita et al described expression of CD30 in 21 of 91 Japanese patients with ATLL.^7^ Like our case, patients in their study presented with lymphomatous variant of ATLL, with only rare cases showing leukemic disease. As a nonspecific activation marker, CD30 may be expressed by a number of non‐Hodgkin lymphoma (NHL) subtypes including ATLL, which should be considered in the differential diagnosis of CD30‐positive lymphoproliferative disorders. Moreover, the availability of brentuximab vedotin, a targeted anti‐CD30 monoclonal antibody‐based therapy, mandates assessment of CD30 expression in T‐cell NHL. Also observed in our case was PD‐1 expression by tumor cells, a feature that may provide a rationale for immune checkpoint inhibitor treatment, which to date has shown variable results in phase 2 trials of ATLL.^8^, ^9^

## CONFLICT OF INTEREST

Authors have no conflict of interest to declare.

## AUTHOR CONTRIBUTIONS

Both authors (MP and AS): equally contributed to manuscript preparation and submission as follows: MP: contributed to composing text and submission of the manuscript. AS: contributed to reviewing main text body and preparation of the corresponding images.

## ETHICAL APPROVAL

The study was approved by the Institutional Review Board of Partners Healthcare/Mass General Brigham.
